# Formulation and evaluation of ulvan-functionalized cumin oil nanoemulsions for antioxidant, anti-photoaging, and anti-melanogenesis activities in skin cells

**DOI:** 10.1186/s40643-025-00980-8

**Published:** 2025-11-25

**Authors:** Fang-Ling Liu, Shih-Yuan Fang, Hsiang-Wen Chan, Ming-Chih Fang, Meng-Tsan Chiang, Chung-Hsiung Huang

**Affiliations:** Department of Food Science, National Taiwan Ocean University, Keelung, 202301 Taiwan

**Keywords:** Cumin essential oil, Melanogenesis, Nanoemulsion, Photoaging, Reactive oxygen species, Skincare, Ulvan

## Abstract

Growing interest in natural skincare solutions has spotlighted bioactive substances like ulvan and cumin essential oil, valued for their roles in combating oxidative stress and inflammation. In this study, cumin oil-based nanoemulsions with and without ulvan extract (CC@NE and UP-CC@NE) were developed using ultrasonication and evaluated for their potential in protecting against oxidative stress, photoaging, and melanogenesis. The formulations exhibited droplet sizes below 100 nm, low polydispersity indices (PDI < 0.2), and negative zeta potentials (− 4.67 to − 11.90 mV), indicating good stability. In vitro assays demonstrated concentration-dependent antioxidant activity, as well as inhibition of tyrosinase, collagenase, and elastase. Both nanoemulsions maintained over 70% cell viability in L929 fibroblasts at 0.25% concentration. In HaCaT keratinocytes, 0.25% nanoemulsions significantly suppressed the production of reactive oxygen species (ROS) triggered by hydrogen peroxide (H_2_O_2_), while in B16F10 melanoma cells, concentrations above 0.025% reduced cell viability and inhibited melanogenesis, with notable effects observed at 0.01–0.025%. Under ultraviolet A (UVA) irradiation, 0.1% nanoemulsions improved cell viability and reduced ROS levels, indicating anti-photoaging effects. Notably, the ulvan extract-containing formulation (UP-CC@NE) consistently outperformed the formulation without ulvan extract, which may suggest potential synergistic effects. These findings highlight the potential of ulvan-functionalized cumin oil nanoemulsions as effective, natural agents for skin protection.

**Graphic abstract:**

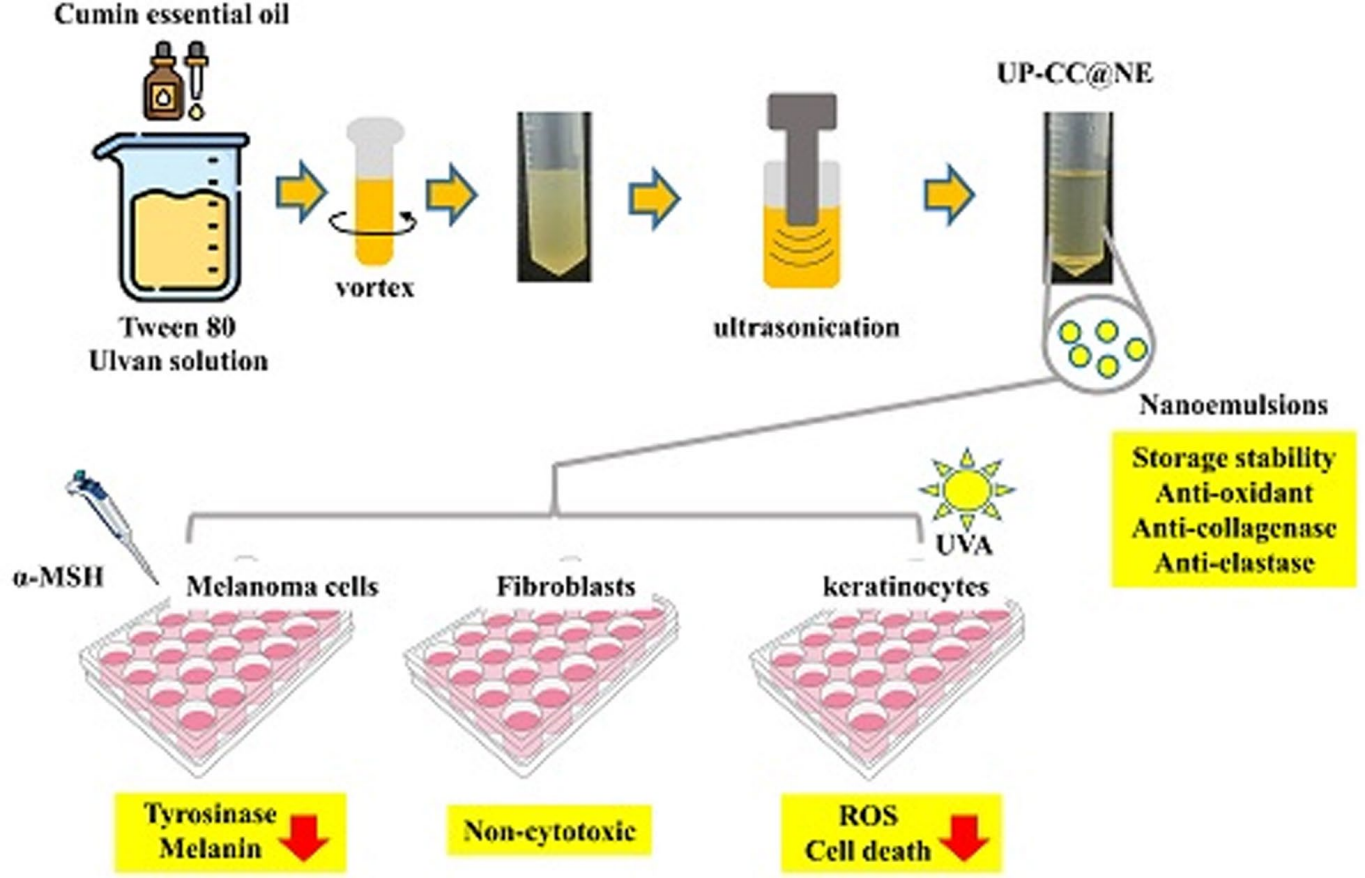

## Introduction

As the body's largest organ, the skin plays a vital role in serving as a protective barrier against physical, chemical, and biological stressors from the external environment. Beyond serving as a barrier, it helps maintain internal homeostasis by preventing dehydration and shielding deeper tissues from harmful stimuli. It is composed of three primary layers, including the epidermis, dermis, and hypodermis, with the epidermal-dermal junction forming a key structural and functional interface. Within the epidermis, keratinocytes are the dominant cell type, producing structural proteins and lipids that maintain skin integrity. In contrast, melanocytes are responsible for producing melanin, the pigment that gives skin its color and helps protect against damage from ultraviolet (UV) radiation (McGrath and Uitto 2023). Skin aging is influenced by internal factors such as genetic regulation and hormonal fluctuations, alongside external elements like environmental exposure. Among these, UV radiation is a major driver of premature skin aging, commonly referred to as photoaging. Among the different types of UV radiation, UVA rays penetrate deeper into the skin. They mainly cause damage by generating reactive oxygen species (ROS), which break down key components of the dermal matrix, such as collagen and elastin. This process leads to visible signs of skin aging, including wrinkles, loss of elasticity, and changes in pigmentation (Lee et al. 2021).

Natural plant-derived compounds are increasingly being explored for skin protection due to their antioxidant and anti-inflammatory potential. *Cuminum cyminum* (cumin), widely used in food and traditional remedies, has demonstrated significant therapeutic potential, including antimicrobial, anti-diabetic, and anti-inflammatory activities (Javadi et al. 2021; Nirmala et al. 2020; Raghuvanshi et al. 2025; Siraj et al. 2023). The essential oil of cumin exhibits strong antioxidant activity by neutralizing ROS such as hydroxyl and peroxide radicals and inhibiting lipid peroxidation, as evidenced by various assays including DPPH (Asgari et al. 2021; El-Ghorab et al. 2010; Keerthiga et al. 2019). Anti-inflammatory effects of cumin have been linked to its flavonoid content, which can modulate key enzymes like inducible nitric oxide synthase and cyclooxygenase-2 (Kang et al. 2019; Singh et al. 2021). Furthermore, cumin essential oil has shown promise in reducing melanin production and tyrosinase activity in melanocyte models, indicating its potential role in regulating pigmentation (Wang et al. 2021). Despite these bioactivities, the direct use of essential oils in skincare is often limited due to their volatility, poor water solubility, and susceptibility to degradation by light, heat, and oxygen (Turek and Stintzing 2013). To overcome these drawbacks, nanoemulsion technology has been employed as a delivery system to enhance the stability and bioavailability of essential oils (Che Marzuki et al. 2019). Nanoemulsions are systems made up of tiny oil droplets dispersed in water, stabilized by surfactants. Their small droplet size (typically 10–200 nm) allows for better dispersion, enhanced absorption, and protection of the active compounds from environmental damage (Wilson et al. 2022).

In addition to plant oils, algal polysaccharides have emerged as effective natural bioactives for dermal applications. Ulvan is a sulfated polysaccharide extracted from green seaweed of the Ulva genus. It contains many functional groups and has shown significant antioxidant, anti-inflammatory, and immunomodulatory effects (Cadar et al. 2025). Its free radical scavenging ability, along with lipid peroxidation inhibition and activation of antioxidant enzymes, contributes to its protective effects on cells under oxidative stress (Cadar et al. 2025; Qi et al. 2005). The biological activity of ulvan appears to be influenced by its molecular mass, with lower molecular mass fractions exhibiting greater antioxidant potential (Qi et al. 2005). In addition, ulvan also modulates inflammatory response and promotes hyaluronic acid production, supporting skin hydration and elasticity (Adrien et al. 2017; Kidgell et al. 2020). Limited research exists on the impact of ulvan on melanin, but similar sulfated polysaccharides like fucoidan inhibit melanogenesis by blocking tyrosinase (Wang et al. 2020).

Nanoemulsions are increasingly employed in topical formulations for their ability to improve skin delivery of both hydrophilic and hydrophobic compounds. Their beneficial physical traits, such as large surface area, clear optical transparency, and lasting stability, improve compound solubility, skin absorption, and effectiveness (Wilson et al. 2022). Prior research has demonstrated the utility of nanoemulsions incorporating plant-based oils, including cumin oil, for improved skin permeation and bioactivity (Morteza-Semnani et al. 2022; Silva et al. 2013). Nevertheless, the full skincare potential of nanoemulsions co-delivering cumin essential oil and algal polysaccharides like ulvan has not been comprehensively evaluated (Hidayat and Fakih 2021; Javadi et al. 2021; Morteza-Semnani et al. 2022; Nirmala et al. 2020; Raghuvanshi et al. 2025; Rostami et al. 2018; Siraj et al. 2023; Syapitri et al. 2022). Due to the growing need for safe and natural skincare, this study develops nanoemulsions with cumin essential oil, with and without ulvan extract, and examines their physical properties and biological effects. Specifically, the formulations were evaluated for their antioxidant capacity, anti-photoaging efficacy, and melanogenesis-inhibitory activity in relevant skin cell models.

## Materials and methods

### Materials

Cumin essential oil and ulvan extract were obtained from AROMARUTH (Kaohsiung, Taiwan) and SWEET TOWN ENTERPRISE CORP. (Hualien, Taiwan), respectively. Cell lines including L929 murine fibroblasts, B16F10 murine melanoma cells, and HaCaT human keratinocytes were acquired from the Bioresource Collection and Research Center (Hsinchu, Taiwan). All analytical-grade chemicals were purchased from Sigma-Aldrich (St. Louis, MO, USA) or PanReac AppliChem (Darmstadt, Germany), while cell culture media and supplements were provided by Thermo Fisher Scientific (Waltham, MA, USA).

### Compositional analysis of ulvan and cumin essential oil

Biochemical constituents of ulvan, including total sugar, uronic acid, sulfate content, phenolics, proteins, and peptides, were analyzed using spectrophotometric and colorimetric methods as described in previous protocols (Chen et al. 2007; Dodgson and Price 1962; DuBois et al. 1956; Miller 1959; Oktay et al. 2003; Slinkard and Singleton 1977). Molecular mass distribution and chemical structure of ulvan extract were determined through high-performance liquid chromatography (HPLC) and Fourier-transform infrared spectroscopy (FTIR), following established methodologies (Ou et al. 2023). Likewise, the chemical profile and functional groups of cumin essential oil were assessed using gas chromatography-mass spectrometry (GC–MS) and FTIR analysis, according to prior studies (Li et al. 2013; Taylan et al. 2021).

### Nanoemulsion preparation and characterization

Nanoemulsions were prepared by homogenizing 40 μL of cumin oil with 180 μL of Tween 80, followed by dropwise addition to 780 μL phosphate-buffered saline (PBS, pH 7.4), with or without ulvan extract (final concentration: 25 mg/mL), based on a previously reported formulation approach with minor modification (Lin et al. 2024). The mixture was stirred until uniform and then subjected to ultrasonication (W-380 ultrasonic processor, 20 kHz; Heat Systems, USA) for 20 min in an ice bath to obtain nanoscale emulsions. No additional pH adjustments were made, and both nanoemulsion formulations had pH values ranging from 5.35 to 5.6. Transmission electron microscopy (TEM; Hitachi HT 7700, Tokyo, Japan) was used to visualize droplet morphology. Particle size, polydispersity index (PDI), and zeta potential were measured using dynamic light scattering (Zetasizer Nano ZS, Malvern Instruments, UK). Emulsion stability was evaluated over 8 weeks under storage at 4 °C, 25 °C, and 37 °C by monitoring size, zeta potential, and PDI at weekly intervals.

### Antioxidant and enzyme inhibitory activities

The antioxidant properties of nanoemulsions were determined using assays for DPPH radical scavenging, Fe^2^⁺ chelating activity, and reducing power, following protocols described in prior research (Campolo et al. 2020; Manap et al. 2021; Slominski et al. 2022; Zhang and McClements 2018; Zhao et al. 2022). Tyrosinase inhibition was also evaluated spectrophotometrically. Inhibitory effects on collagenase and elastase were assessed using commercial enzyme assay kits: EnzChek™ Collagenase/Gelatinase Assay Kit (E12055) and EnzChek™ Elastase Assay Kit (E12056), respectively (Invitrogen™, Thermo Fisher Scientific), following the manufacturer’s instructions.

### Cell viability and cellular assays

Cell viability was assessed using the MTT assay as per ISO 10993–5 standards and previous studies (de Sá et al. 2020). L929, HaCaT, and B16F10 cells were seeded at 1 × 10^5^ cells/mL and cultured for 24 h. After replacing the medium, nanoemulsions were added at concentrations ranging from 0 to 1% (v/v) and incubated for another 24 h prior to viability assessment.

To evaluate intracellular ROS levels in HaCaT cells, cells were seeded at 2 × 10^4^ cells/well in 96-well plates and pre-treated with various concentrations of nanoemulsions in phenol red-free medium for 24 h (Han et al. 2021; Piras et al. 2024). Cells were then stained with 10 µM H_2_DCFDA for 30 min, washed with PBS, and exposed to 1 mM hydrogen peroxide (H_2_O_2_). ROS generation was monitored by measuring DCF fluorescence (excitation: 490 nm; emission: 520 nm) at 15-min intervals over 75 min.

For assessing melanin biosynthesis, B16F10 cells (5 × 10^3^ cells/mL) were stimulated with 100 nM α-MSH for 24 h, followed by treatment with nanoemulsions for 72 h (Lee et al. 2006). Extracellular melanin content was determined by measuring absorbance at 405 nm in the culture medium. For intracellular melanin, cells were lysed using 1% Triton X-100, heated, and centrifuged; pellets were resuspended in NaOH and absorbance at 405 nm was recorded. Results were expressed relative to α-MSH-stimulated controls (set as 100%).

### Evaluation of anti-photoaging effects under UVA exposure

HaCaT cells were seeded at 5 × 10^4^ cells/mL and incubated for 24 h. Following removal of culture medium, 0.1% nanoemulsion was applied and incubated either before or after UVA exposure. For irradiation, PBS replaced the medium, and cells were exposed to UVA at a total dose of 10 J/cm^2^. Post-exposure, cells were replenished with fresh medium and incubated for 24 h. ROS production was quantified using the H_2_DCFDA assay, and cell viability was analyzed via MTT assay and propidium iodide (PI) staining.

### Statistical analysis

Three independent biological experiments were conducted, each yielding consistent results. The data presented in the manuscript represent one representative biological experiment. Within each experiment, technical triplicates were performed. One-way ANOVA was used to evaluate overall differences among experimental groups. When significant differences were observed, Tukey’s post-hoc test was performed to conduct pairwise comparisons between the control group and each treatment group (SigmaPlot v14, Systat Software Inc.). For antioxidant, anti-tyrosinase, anti-collagenase, and anti-elastase activities of CC@NE and UP-CC@NE, statistical comparisons were made between UP-CC@NE and CC@NE at the same concentration. A *p* value of < 0.05 was considered statistically significant for all tests.

## Results

### Characterization of ulvan and cumin essential oil

**Fig. 1:**
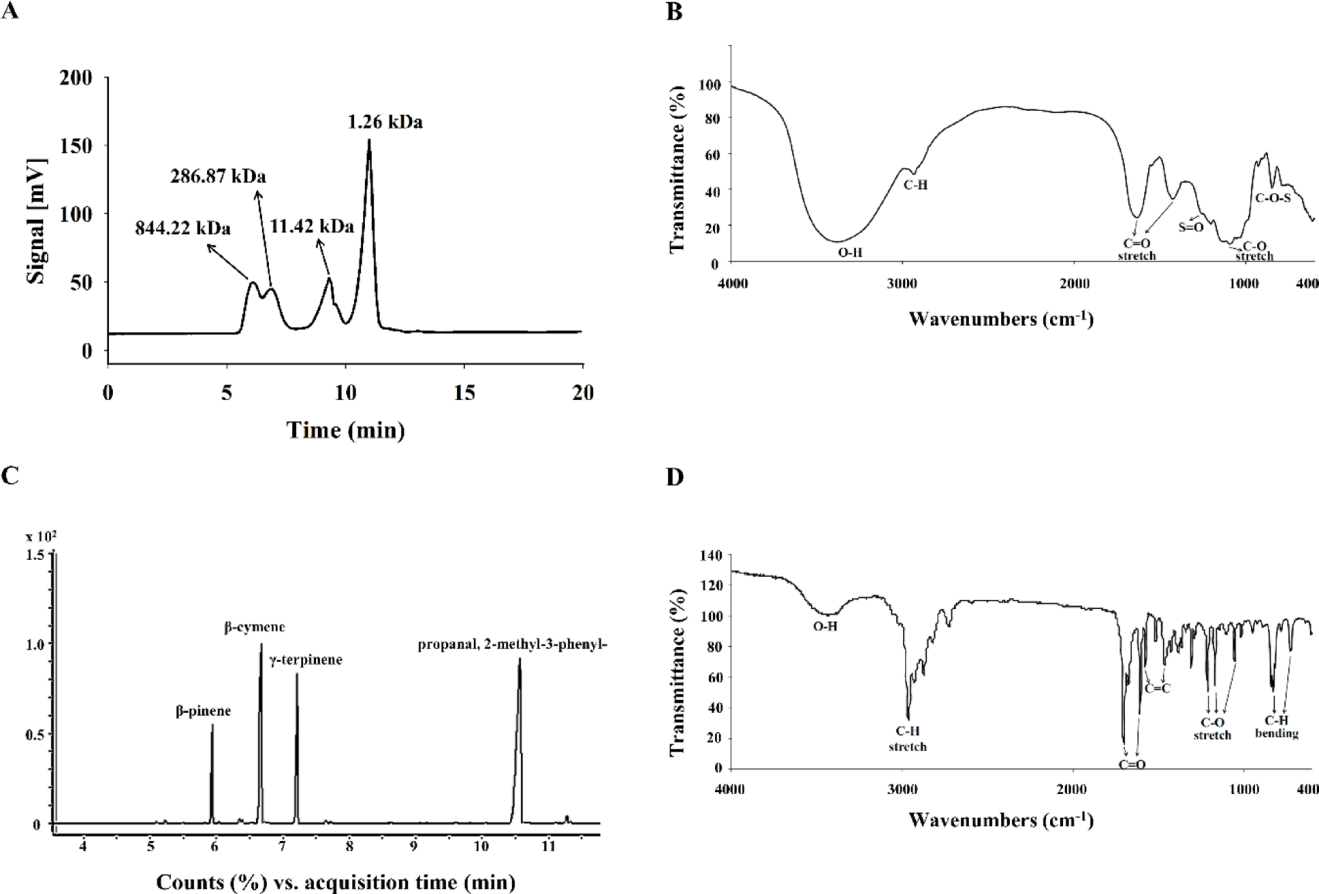
Characterization of ulvan extract and cumin essential oil. **A** HPLC chromatograms of ulvan extract analyzed by polysaccharide size exclusion column (SB-804 HQ). **B** ATR-FTIR chromatograms of ulvan extract. Wave range: 600–4000 cm^−1^; standard parameters: 4 cm^−1^, 32 scans. **C** Total ion chromatogram of cumin essential oil analyzed by GC–MS. **D** ATR-FTIR chromatograms of cumin essential oil. Wave range: 600–4000 cm^−1^; standard parameters: 2 cm^−1^, 36 scans

Ulvan extract composition analysis revealed a total sugar content of 53.19%, with substantial amounts of uronic acid (40.92%) and sulfated groups (25.99%). Minor components included total soluble proteins (13.46%), peptides (4.82%), and polyphenols (0.08%) (Table 1). HPLC profiling indicated four distinct molecular mass peaks at 844.22, 286.87, 11.42, and 1.26 kDa, suggesting a heterogeneous polysaccharide structure spanning high to low molecular mass (Fig. 1A). FTIR spectra of ulvan extract displayed absorption bands at 3269, 1607, 1419, 1252, 1023, and 845 cm^−1^, consistent with hydroxyl groups, carboxyl and sulfate groups, and glycosidic linkages (Fig. 1B) (Barakat et al. 2022).

**Table 1:** Chemical composition of ulvan extract

Compounds	Contents (%)
Total sugar	53.2 ± 1.1
Uronic acid	40.1 ± 0.4
Sulfate	26.0 ± 0.6
Total phenol	0.1 ± 0
Protein	13.5 ± 0.4
Peptide	4.8 ± 0.1

Each value is expressed as the mean ± standard deviation (n = 3)

GC–MS analysis of cumin essential oil identified seven primary constituents, with propanal, 2-methyl-3-phenyl- (46.97%), β-cymene (27.24%), γ-terpinene (15.36%), and β-pinene (8.35%) as major components (Table 2, Fig. 1C). FTIR analysis further confirmed characteristic functional groups, with peaks associated with C–H stretching (2959 cm^−1^), carbonyl (1701, 1674 cm^−1^), aromatic C=C (1575, 1461 cm^−1^), and C–O bond vibrations (1211, 1169, 1064 cm−^1^). Aromatic C–H bending was observed near 839–815 cm^−1^, indicating the presence of various phenolic and aromatic constituents (Fig. 1D) (Osanloo et al. 2024; Ranjbar et al. 2023).

**Table 2:** Composition of cumin essential oil

Compounds	Contents (%)
β-Pinene	8.4 ± 0.1
α-Phellandrene	0.7 ± 0
β-Cymene	27.2 ± 0.9
γ-Terpinene	15.4 ± 0.5
Propanal, 2-methyl-3-phenyl-	47.0 ± 1.6
2-Caren-10-al	0.9 ± 0
β-Gurjunene	0.6 ± 0

Each value is expressed as the mean ± standard deviation (n = 3)

### Physicochemical properties and stability of nanoemulsions

**Fig. 2:**
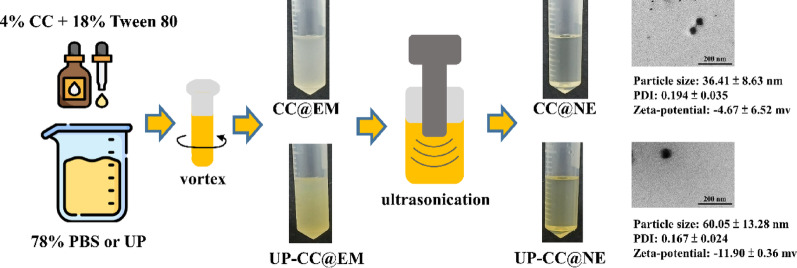
Development and characterization of cumin essential oil-nanoemulsions. A schematic representation of the preparation of CC@NE and UP-CC@NE, along with their characteristic morphology as observed through TEM. Each value of droplet size, PDI, and zeta potential of CC@NE and UP-CC@NE is expressed as the mean ± standard deviation (n = 3)

The optimized nanoemulsion formulation comprised 40 μL cumin oil, 180 μL Tween 80, 780 μL PBS, and ulvan extract at 25 mg/mL. This mixture, processed by ultrasonication, yielded a transparent and stable oil-in-water nanoemulsion (Fig. 2). Before emulsification, the formulations appeared turbid; post-sonication, clarity increased markedly. The incorporation of ulvan extract resulted in reduced droplet size and a more negative zeta potential, while maintaining a low PDI, indicating uniformity in droplet distribution. Both emulsions exhibited droplet sizes under 100 nm. Storage tests conducted over eight weeks at 4 °C, 25 °C, and 37 °C revealed minimal variations in size, PDI, and zeta potential, confirming the long-term physical stability of nanoemulsions (Table 3).

**Table 3:** Change in particle size, polydispersity index, and zeta potential of CC@NE and UP-CC@NE at 4 °C, 25 °C and 37 °C

	CC@NE	Week 0	Week 2	Week 4	Week 6	Week 8
4 °C	Particle size (nm)	27.5 ± 3.6^bA^	36.8 ± 1.8^aA^	20.2 ± 0.0^cA^	19.9 ± 0.4^cA^	15.7 ± 1.3^dA^
	PDI	0.2 ± 0.0^Aa^	0.2 ± 0.0^aB^	0.2 ± 0.1^aA^	0.2 ± 0.1^aA^	0.2 ± 0.1^aA^
	Zeta-potential (mV)	− 6.5 ± 1.2^cB^	− 3.7 ± 0.2^bA^	− 3.5 ± 0.3^bA^	− 1. 6 ± 0.8^aA^	− 1.5 ± 0.5^aB^
25 °C	Particle size (nm)	30.4 ± 2.7^aA^	21.3 ± 0.4^bA^	13.4 ± 1.2^cA^	20.0 ± 2.4^bA^	11.2 ± 0.9^cB^
	PDI	0.2 ± 0.0^aA^	0.2 ± 0.0^aA^	0.2 ± 0.1^aA^	0.2 ± 0.1^aA^	0.2 ± 0.0^aA^
	Zeta-potential (mV)	− 0.9 ± 0.0^aA^	− 1.6 ± 1.0^aA^	− 0.9 ± 0.4^aA^	− 0.3 ± 0.3^aA^	− 0.9 ± 0.2^aAB^
37 °C	Particle size (nm)	19.9 ± 0.2^aB^	20.3 ± 0.9^aB^	21.9 ± 7.2^aA^	16.9 ± 1.0^aA^	13.2 ± 1.3^aB^
	PDI	0.2 ± 0.0^aA^	0.2 ± 0.0^aAB^	0.2 ± 0.0^aA^	0.2 ± 0.0^aA^	0.2 ± 0.0^aA^
	Zeta-potential (mV)	− 2.7 ± 1.2^aA^	− 4.3 ± 2.7^aA^	− 2.9 ± 1.8^aA^	− 1.0 ± 0.2^aA^	− 0.5 ± 0.2^aA^

	UP-CC@NE	Week 0	Week 2	Week 4	Week 6	Week 8
4 °C	Particle size (nm)	70.2 ± 8.7^aA^	61.3 ± 5.1^aA^	58.5 ± 4.5^aA^	59.0 ± 1.3^aA^	61.2 ± 6.8^aA^
	PDI	0.0 ± 0.0^aA^	0.2 ± 0.0^aA^	0.1 ± 0.0^aA^	0.1 ± 0.1^aA^	0.1 ± 0.0^aA^
	Zeta-potential (mV)	− 7.6 ± 0.7^bA^	− 6.7 ± 0.8^bB^	− 2.2 ± 1.7^aA^	− 4.1 ± 1.3^aA^	− 3.5 ± 1.4^aA^
25 °C	Particle size (nm)	54.8 ± 4.2^bcA^	63.6 ± 3.4^aA^	63.9 ± 4.0^aA^	62.5 ± 4.4^aA^	53.9 ± 5.7^cA^
	PDI	0.2 ± 0.1^aA^	0.2 ± 0.0^aA^	0.2 ± 0.0^aA^	0.2 ± 0.1^aA^	0.2 ± 0.0^aA^
	Zeta-potential (mV)	− 7.7 ± 1.4^aA^	− 6.3 ± 0.7^aB^	− 4.5 ± 0.8^aB^	− 5.8 ± 3.0^aA^	− 6.9 ± 1.2^aB^
37 °C	Particle size (nm)	33.4 ± 9.8^aB^	32.6 ± 2.7^aB^	31.1 ± 6.8^aB^	29.7 ± 2.6^aB^	29.5 ± 3.2^aB^
	PDI	0.2 ± 0.1^aA^	0.2 ± 0.0^aA^	0.1 ± 0.1^aA^	0.2 ± 0. 1^aA^	0.2 ± 0.0^aA^
	Zeta-potential (mV)	− 4.5 ± 2.4^aA^	− 4.4 ± 0.8^aA^	− 5.1 ± 0.7^aB^	− 5.8 ± 2.6^aA^	− 3.9 ± 0.3^aA^

Each value is expressed as the mean ± standard deviation (n = 3)

^a^^−^^d^Different letters indicate significantly different values (*p* < 0.05) in various storage time with the same temperature

^A^^−^^B^Different letters indicate significantly different values (*p* < 0.05) in various temperature with the same storage time

### Antioxidant capacity and enzymatic inhibition

**Fig. 3:**
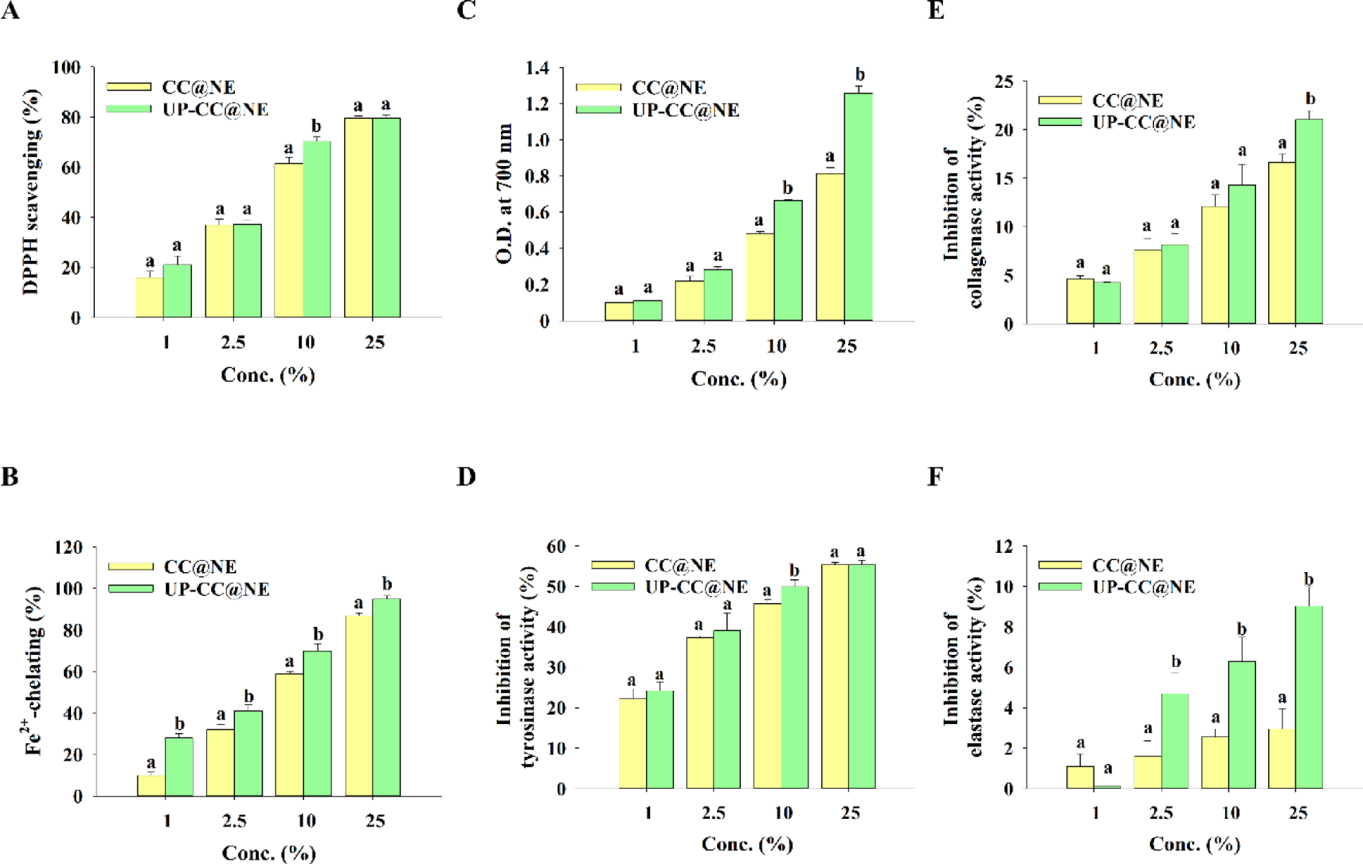
Antioxidant, anti-tyrosinase, anti-collagenase and anti-elastase activities of CC@NE and UP-CC@NE. **A** The DPPH radicals scavenging activities, **B** ferrous ion-chelating activities, **C** reducing power, **D** inhibition of tyrosinase activities, **E** inhibition of collagenase activities, and **F** inhibition of elastase activities of CC@NE amd UP-CC@NE. Each value is expressed as the mean ± standard deviation (n = 3). Statistical comparisons were made between UP-CC@NE and CC@NE at the same concentration, with different letters denoting significantly different values (p < 0.05)

The nanoemulsions demonstrated dose-dependent antioxidant activity in assays for DPPH radical scavenging, Fe^2^⁺ chelation, and reducing power. Additionally, they inhibited tyrosinase, collagenase, and elastase enzymes in a concentration-dependent manner across a 1–25% range, with UP-CC@NE exhibiting superior efficacy compared to CC@NE (Fig. 3).

### Cytotoxicity and ROS scavenging in fibroblasts and keratinocytes

**Fig. 4:**
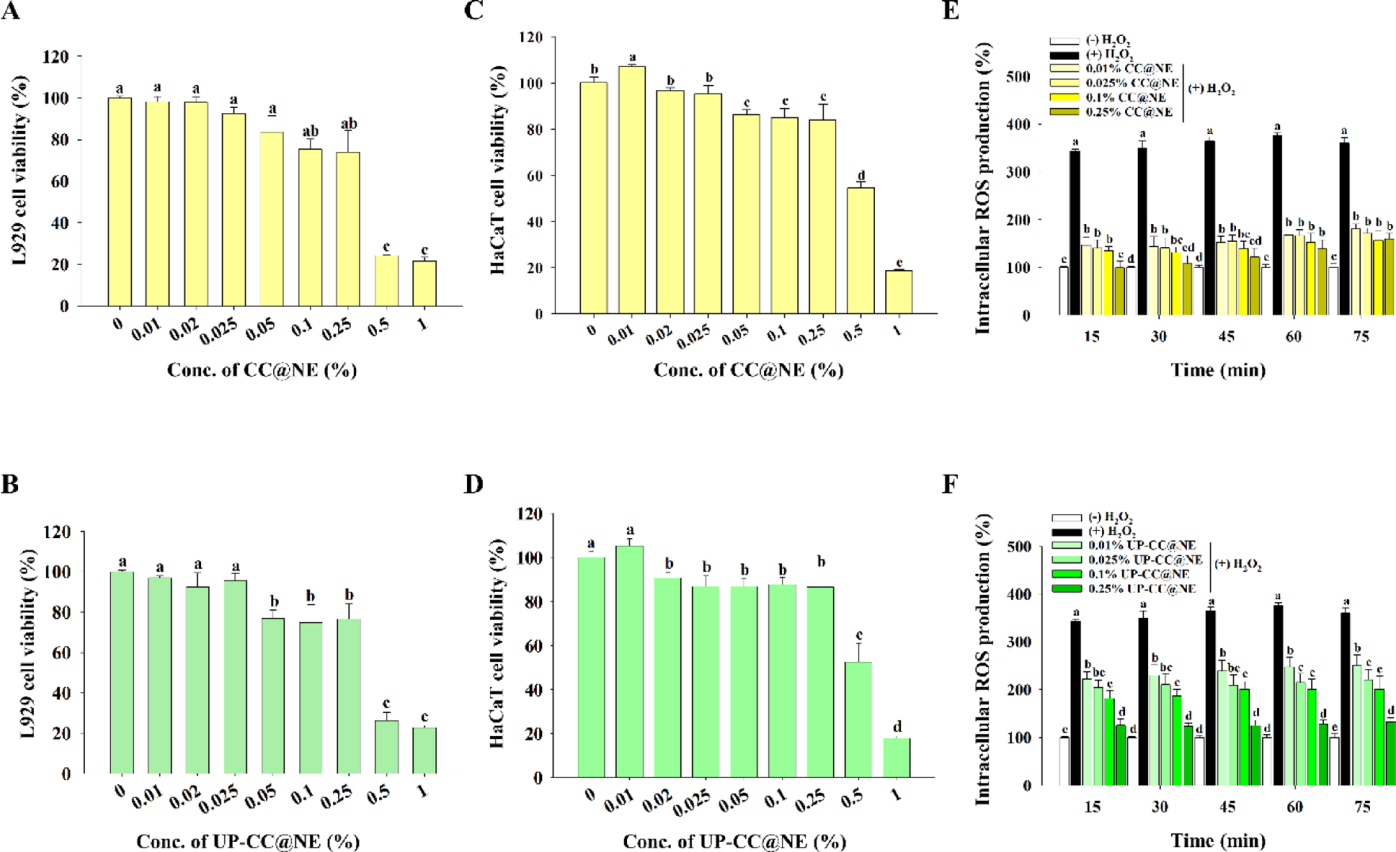
Impact of nanoemulsions on cell viability and H_2_O_2_-induced intracellular ROS production. **A**, **B** L929 cells and **C**, **D** HaCaT cells were incubated in the presence of either CC@NE or UP-CC@NE (0, 0.01, 0.02, 0.025, 0.05, 0.1, 0.25, 0.5 or 1%) for 24 h, and cell viability was assessed. The viability of untreated cells was designed as 100%. **E**, **F** HaCaT cells were incubated with CC@NE or UP-CC@NE (0, 0.01, 0.025, 0.1, or 0.25%) for 24 h, followed by stimulation with 1 mM H_2_O_2_. Intracellular ROS production was assessed by tracking the fluorescence intensity of DCF every 15 min for a duration of 75 min. The data are presented as the mean ± standard deviation (n = 3), with different letters denoting significantly different values (p < 0.05)

Cytotoxicity assays using L929 fibroblasts showed that cell viability remained above 70% at concentrations ≤ 0.25% nanoemulsion (Fig. 4A, B), meeting ISO 10993-5 standards (de Sá et al. 2020). A similar threshold was observed in HaCaT keratinocytes, with significant cytotoxicity appearing only at concentrations ≥ 0.5% (Fig. 4C, D). Based on these results, concentrations below 0.25% were selected for subsequent experiments in HaCaT cells to ensure testing under non-cytotoxic conditions. To evaluate ROS-scavenging capacity, HaCaT cells were pretreated with nanoemulsions, then exposed to 1 mM H_2_O_2_. Intracellular ROS levels, assessed via H_2_DCFDA fluorescence, were significantly attenuated in nanoemulsion-treated cells with effects increasing according to concentration (Fig. 4E, F). These findings suggest both formulations protect skin cells from oxidative damage.

### Inhibition of α-MSH-induced melanogenesis

**Fig. 5:**
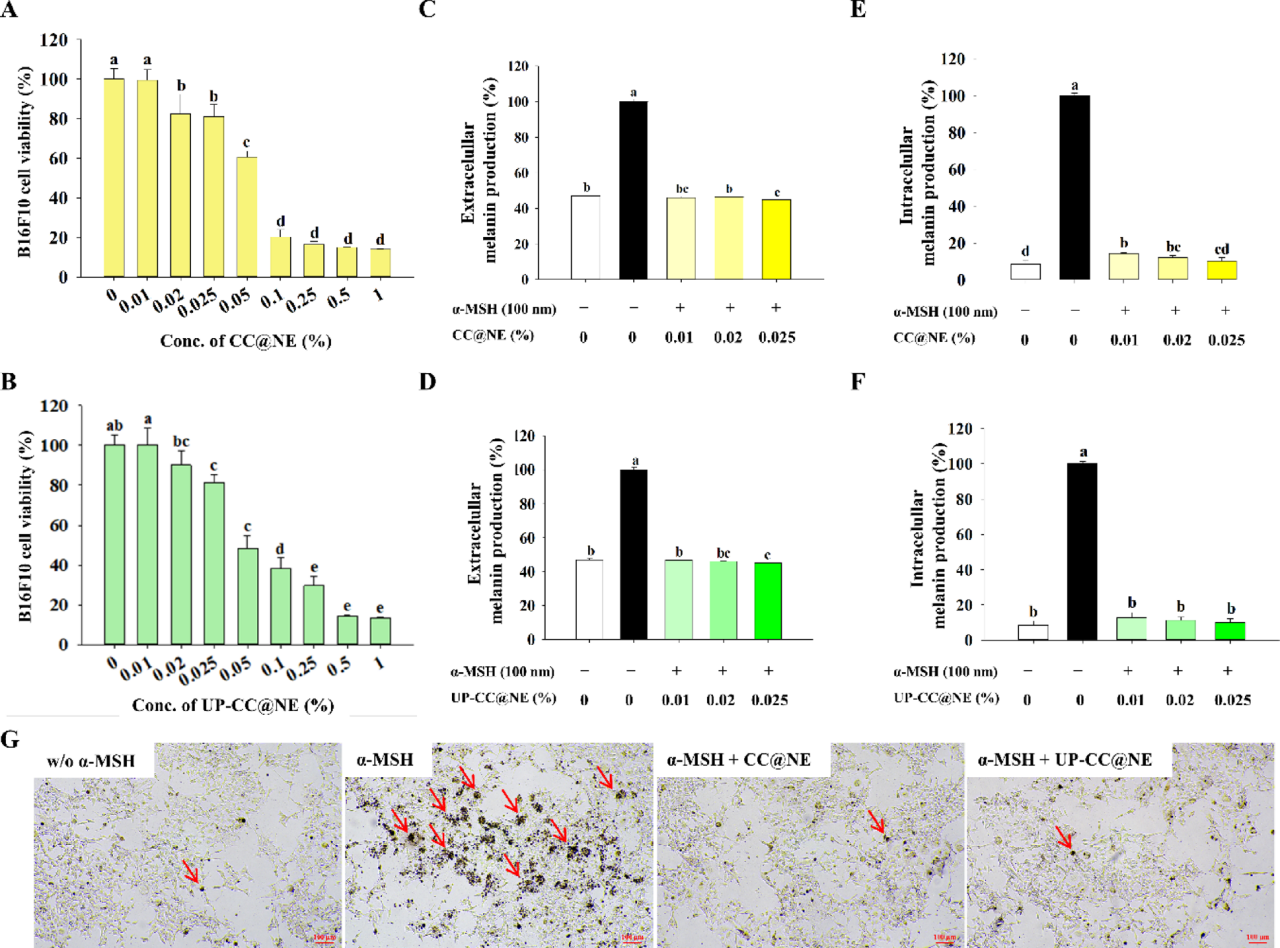
Inhibitory effects of nanoemulsions on melanogenesis. B16F10 cells were incubated with varying concentrations of **A** CC@NE or **B** UP-CC@NE (0, 0.01, 0.02, 0.025, 0.05, 0.1, 0.25, 0.5 or 1%) for 24 h, and cell viability was assessed. B16F10 cells were treated with 100 nM α-MSH with varying concentrations of nanoemulsions (0, 0.01, 0.02, or 0.025%), and the **C**, **D** extracellular and **E**, **F** intracellular melanin production were assessed as described in the Material and Methods. The data are presented as the mean ± standard deviation (n = 3), with different letters denoting significantly different values (*p* < 0.05). **G** Representative images of B16F10 cells treated without or with α-MSH and nanoemulsions (0.01%) observed under a microscope at 40 × magnification. The red arrows indicate melanin granules

B16F10 melanoma cells exposed to nanoemulsions showed reduced viability at higher concentrations, with > 70% viability retained at ≤ 0.025% (Fig. 5A, B), Based on these findings, a series of concentrations below 0.025% was selected for further experiments to evaluate the effects of the nanoemulsion on melanin suppression under non-cytotoxic conditions. This range allowed for the investigation of potential concentration-dependent responses while ensuring cell viability remained within the acceptable threshold. Upon α-MSH stimulation, melanin production significantly increased, whereas nanoemulsion treatment at 0.01–0.025% substantially reduced both extracellular and intracellular melanin levels. Specifically, extracellular melanin dropped from 100% in the α-MSH group to 46.92% in the non-stimulated group and even lower in treated groups (Fig. 5C, D). Intracellular melanin similarly declined from 100% (α-MSH group) to below 20% in treated cells (Fig. 5E, F). Microscopic observation confirmed reduced pigment granules in cells treated with CC@NE and UP-CC@NE (Fig. 5G).

### Protective effects against UVA-induced damage

**Fig. 6:**
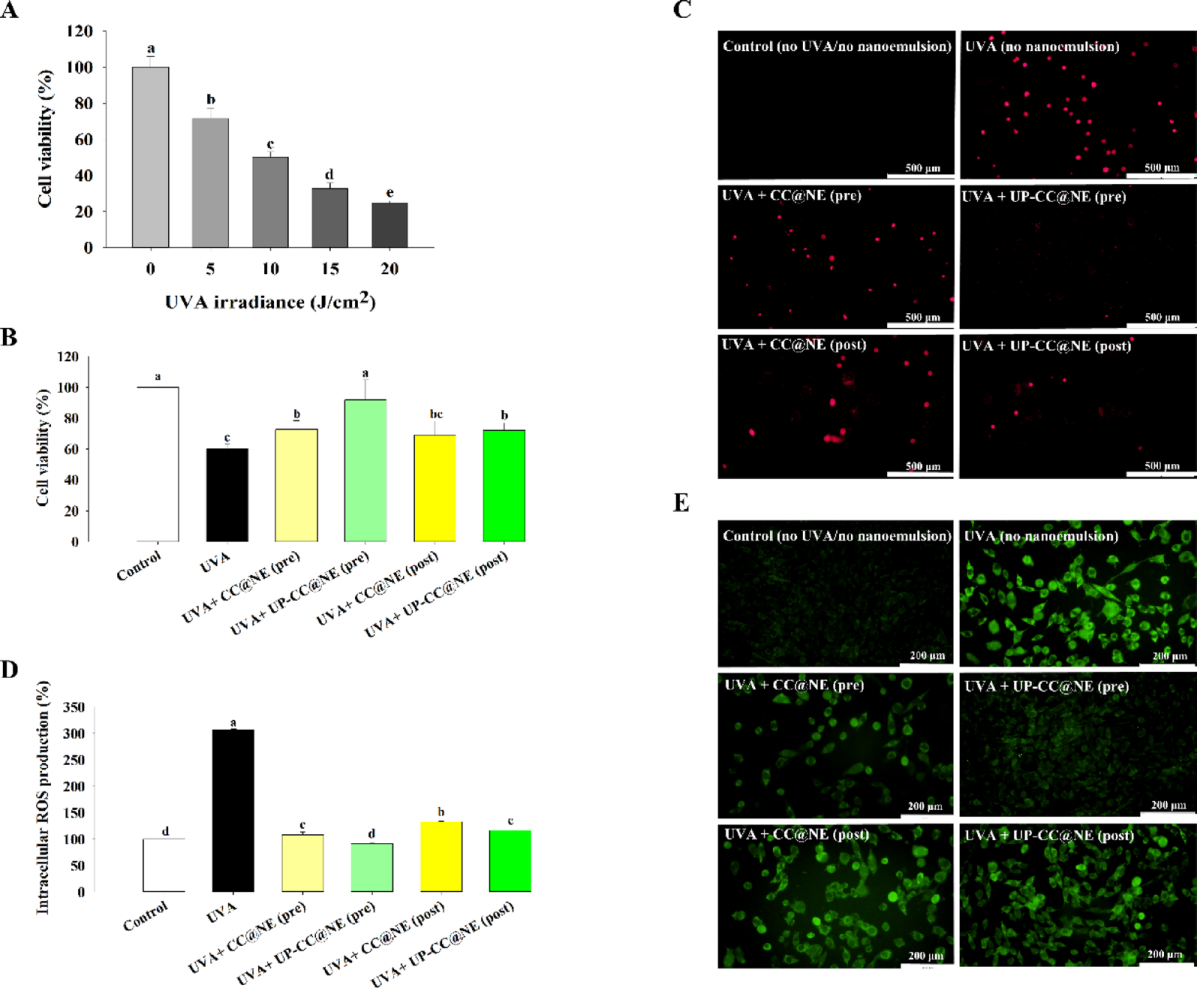
Effects of nanoemulsions on attenuating UVA-induced oxidative damage. **A** HaCaT cells were exposed to varying doses of UVA (0–20 J/cm^2^), and cell viability was assessed. The viability of cells without UVA exposure was designed as 100%. HaCaT cells were incubated with either CC@NE or UP-CC@NE (0.1%) before or after UVA exposure (10 J/cm^2^). Cell viability was evaluated by **B** MTT assay and **C** propidium iodide (PI) staining. Intracellular ROS production was measured by **D** H_2_DCFDA assay and **E** observed under a fluorescence microscope as described in the Material and Methods. DCF fluorescence was detected using an excitation wavelength of 490 nm and an emission wavelength of 520 nm. The control group consists of cells that were neither exposed to UVA irradiation nor treated with the nanoemulsion. The data are presented as the mean ± standard deviation (n = 3), with different letters denoting significantly different values (*p* < 0.05)

To establish UVA sensitivity, HaCaT cells were irradiated with 5–20 J/cm^2^. Cell viability decreased with increasing UVA dose, with 10 J/cm^2^ reducing viability to 50%, and was selected for subsequent anti-photoaging evaluation (Fig. 6A). Both pre- and post-treatment with 0.1% nanoemulsions significantly enhanced cell viability following UVA exposure, particularly in the ulvan extract-containing formulation (Fig. 6B). Propidium iodide staining supported these findings, showing fewer dead cells in nanoemulsion-treated groups compared to UVA-only controls (Fig. 6C).

ROS levels in UVA-exposed cells were significantly elevated, but treatment with either nanoemulsion markedly reduced ROS accumulation (Fig. 6D). Microscopy confirmed reduced fluorescence intensity, with UP-CC@NE pretreatment yielding the most pronounced ROS inhibition (Fig. 6E). These results suggest both nanoemulsions, particularly the ulvan extract-enriched variant, confer protective effects against UVA-induced oxidative stress and cellular damage.

## Discussion

In this study, we successfully created a nanoemulsion with cumin essential oil (CC@NE) and an improved version containing ulvan extract (UP-CC@NE) for skincare. These formulations targeted oxidative stress, UV damage, and hyperpigmentation. Both nanoemulsions showed good physical properties, biological activity, and stability, especially the one with ulvan extract, making them promising natural skincare options.

Lipid-based nanocarriers, including nanoemulsions, are well-regarded for enhancing the delivery and bioavailability of hydrophobic compounds by protecting them from chemical degradation and facilitating their dispersion in aqueous environments (Danaei et al. 2018). Structurally, nanoemulsions are formed by dispersing oil droplets within an aqueous phase, where surfactants are used to maintain their stability. Their optical transparency typically increases as droplet size decreases below the wavelength of visible light, due to reduced light scattering (Zhang and McClements 2018). In this study, ultrasonication markedly improved emulsion clarity, indicating effective droplet size reduction. The resulting nanoemulsions exhibited droplet sizes of approximately 36.41 nm and a PDI below 0.25, indicating a narrow particle size distribution and good physical stability. After ulvan extract incorporation, droplet size increased to 60.05 nm, which was expected due to surface modification, but the PDI remained under 0.25, further confirming stable dispersion. The nanoemulsions retained clarity and structural integrity over eight weeks of storage at 4, 25, and 37 °C, indicating strong long-term physical stability. Although minor fluctuations in droplet size were observed, such as the decrease in CC@NE at 4 °C, these variations fall within expected experimental variability and do not reflect structural degradation. Both CC@NE and UP-CC@NE maintained consistently low PDI (< 0.25), with no visible signs of aggregation or phase separation, further supporting the stability of the formulations. Despite relatively low zeta potential values (–4.67 mV for CC@NE and –11.90 mV for UP-CC@NE), which typically indicated thermodynamic instability (Che Marzuki et al. 2019), the emulsions remained stable throughout storage. This apparent contradiction suggests that steric stabilization was the primary mechanism governing stability in this system. Tween 80, a non-ionic surfactant used in the formulation, likely formed a sterically protective interfacial layer that prevents droplet coalescence, even in the absence of strong electrostatic repulsion (Campolo et al. 2020). The high aqueous content (78%) also facilitated the extension and distribution of surfactant chains, enhancing steric hindrance. Additionally, ulvan extract might contribute to this effect by modifying the droplet surface without compromising dispersion stability. These results support previous studies showing that Tween 80-stabilized emulsions remain stable over time (Lin et al. 2024; Raghuvanshi et al. 2025), confirming that steric stabilization helps maintain nanoemulsion stability during storage.

Comparative analysis with prior research reveals both similarities and unique aspects of the current work. For example, Nirmala et al. demonstrated that cumin oil-based nanoemulsions possessed anticancer and antimicrobial properties, with ultrasonication producing droplet sizes under 100 nm, consistent with our results (Nirmala et al. 2020). Similarly, Javadi et al. developed oil-in-water nanoemulsions for antidiabetic applications using cumin essential oil and observed enhanced stability and bioavailability of encapsulated plant extracts, despite low zeta potential values (Javadi et al. 2021). Our study corroborates these observations and adds new insight by highlighting the contribution of ulvan extract to nanoemulsion stability and bioactivity. Moreover, Katayoun et al. applied cumin oil nanoemulsions in transdermal drug delivery systems, reporting improved skin permeation and therapeutic effects with droplet sizes around 82 nm (Morteza-Semnani et al. 2022). These findings suggest the potential of such systems for delivering polysaccharides like ulvan, although further optimization in the form of nanoemulgels remains an area for future exploration. Similarly, Siraj et al. showed that cumin nanoemulsions boosted antimicrobial activity, especially against drug-resistant bacteria (Siraj et al. 2023). Raghuvanshi et al. used encapsulated cumin oil to extend dairy products’ shelf life, highlighting its potential in both medicine and food industries (Raghuvanshi et al. 2025).

Skin health is closely associated with protection from environmental stressors such as UV radiation and pollution. These factors trigger the formation of ROS, leading to oxidative damage, extracellular matrix degradation, and melanogenesis. Antioxidants play a key role in counteracting such effects (Lee et al. 2021). In the current study, the antioxidant properties of the nanoemulsions were demonstrated through in vitro assays, including DPPH scavenging, ferrous ion chelation, and reducing power tests. A concentration-dependent increase in antioxidant activity was observed. Notably, the ulvan extract-containing formulation exhibited superior antioxidant efficacy, which may be attributed to potential synergistic interactions between ulvan extract and the phenolic compounds in cumin essential oil. While the precise mechanisms underlying this interaction remained to be elucidated, the observed data suggest that both components contributed meaningfully to ROS mitigation. The protective effects of the nanoemulsions were further tested in HaCaT keratinocytes exposed to H_2_O_2_, a common method to mimic oxidative damage caused by hydroxyl radicals through the Fenton reaction (Piras et al. 2024). Both formulations significantly reduced intracellular ROS levels, indicating cytoprotective properties.

Melanin, while essential for UV protection, can lead to hyperpigmentation when overproduced. Its synthesis is tightly regulated by tyrosinase, a key enzyme in melanogenesis (Slominski et al. 2022; Zhao et al. 2022). Tyrosinase inhibition has thus become a target for skin-lightening and anti-hyperpigmentation strategies (Manap et al. 2021). In this study, nanoemulsions exhibited dose-dependent inhibition of mushroom tyrosinase. However, ulvan extract did not enhance this effect, suggesting that its primary role was not in direct enzyme inhibition. The effect of nanoemulsions on melanin production was tested in B16F10 melanoma cells treated with α-MSH, a peptide that increases melanin in response to UV exposure (Dall’Olmo et al. 2023). At concentrations ≤ 0.025%, nanoemulsions reduced both extracellular and intracellular melanin levels without cytotoxicity, suggesting selective activity against melanocytes or melanoma cells, while sparing fibroblasts and keratinocytes. This highlights the potential of the nanoemulsions as depigmenting agents with minimal side effects on normal skin cells.

UVA radiation contributes significantly to photoaging through oxidative stress, extracellular matrix breakdown, and apoptosis in skin cells (Lee et al. 2021). Here, HaCaT cells were exposed to UVA at 10 J/cm^2^, which reduced viability by 50%, a commonly accepted threshold in anti-photoaging research (de Carvalho et al. 2021; Liang et al. 2021). Treatment with nanoemulsions before or after irradiation significantly improved cell viability and reduced ROS generation, with UP-CC@NE showing the most pronounced effects. These results suggest that ulvan extract, rich in sulfated and uronic acid groups (26% and 41%, respectively), may contribute to photoprotection, consistent with other studies highlighting the antioxidant and UV-absorbing properties of algal polysaccharides (Chang et al. 2024).

While the nanoemulsions demonstrated promising physicochemical stability under controlled in vitro conditions, these findings may not fully predict their performance in complex biological environments. The absence of in vivo studies limits our understanding of their safety, efficacy, and biological interactions, such as skin penetration, systemic absorption, and metabolic fate, which are critical for cosmetic or dermatological applications. Therefore, comprehensive in vivo evaluations are necessary to validate the translational potential of these formulations. Additionally, the use of a commercially sourced ulvan extract without further purification presents another methodological limitation. The presence of co-extracted components, including proteins and peptides, may influence the observed bioactivities. As a result, it is difficult to attribute the biological effects solely to ulvan, and this potential confounding should be considered when interpreting the results.

## Conclusion

This study highlights the potential of nanoemulsions combining cumin essential oil and ulvan extract as multifunctional agents for skincare. The formulations exhibited good physical stability and were found to be biocompatible at lower concentrations. They effectively reduced oxidative damage, suppressed melanin production, and offered protection against photoaging. The presence of ulvan extract notably enhanced the antioxidant and photoprotective effects, underscoring its value as a natural bioactive compound within the formulation. Despite the generally low toxicity and promising bioactivity observed, some variability in biological outcomes across different tests suggests that further comprehensive safety evaluations and dose optimization are necessary. Moving forward, in vivo studies and the development of advanced delivery platforms, such as nanoemulgels, will be important to improve the practical applicability of these nanoemulsions. Overall, the results suggest that cumin essential oil and ulvan extract-based nanoemulsions hold considerable promise as natural, safe, and multifunctional ingredients for future dermatological products.

## Data Availability

Data will be made available on request.

## Abbreviations

α-MSH: α-Melanocyte-stimulating hormone
CC@NE: Nanoemulsions without ulvan extract
FTIR: Fourier-transform infrared spectroscopy
GC-MS: Gas chromatography-mass spectrometry
HPLC: High-performance liquid chromatography
MTT: 3-(4,5-Dimethylthiazol-2-yl)-2,5-diphenyltetrazolium bromide
PBS: Phosphate-buffered saline
PDI: Polydispersity index
PI: Propidium iodide
ROS: Reactive oxygen species
TEM: Transmission electron microscopy
UP-CC@NE: Nanoemulsions with ulvan extract
